# Alcohol consumers’ attention to warning labels and brand information on alcohol packaging: Findings from cross-sectional and experimental studies

**DOI:** 10.1186/s12889-017-4055-8

**Published:** 2017-01-26

**Authors:** Inge Kersbergen, Matt Field

**Affiliations:** Department of Psychological Sciences, University of Liverpool, UK and the UK Centre for Tobacco and Alcohol Studies (UKCTAS), Liverpool, L69 7ZA UK

**Keywords:** Alcohol, Alcohol packaging, Eye-tracking, Health warnings, Motivation to reduce drinking, Visual attention

## Abstract

**Background:**

Alcohol warning labels have a limited effect on drinking behavior, potentially because people devote minimal attention to them. We report findings from two studies in which we measured the extent to which alcohol consumers attend to warning labels on alcohol packaging, and aimed to identify if increased attention to warning labels is associated with motivation to change drinking behavior.

**Methods:**

Study 1 (*N* = 60) was an exploratory cross-sectional study in which we used eye-tracking to measure visual attention to brand and health information on alcohol and soda containers. In study 2 (*N* = 120) we manipulated motivation to reduce drinking using an alcohol brief intervention (vs control intervention) and measured heavy drinkers’ attention to branding and warning labels with the same eye-tracking paradigm as in study 1. Then, in a separate task we experimentally manipulated attention by drawing a brightly colored border around health (or brand) information before measuring participants’ self-reported drinking intentions for the subsequent week.

**Results:**

Study 1 showed that participants paid minimal attention to warning labels (7% of viewing time). Participants who were motivated to reduce drinking paid less attention to alcohol branding and alcohol warning labels. Results from study 2 showed that the alcohol brief intervention decreased attention to branding compared to the control condition, but it did not affect attention to warning labels. Furthermore, the experimental manipulation of attention to health or brand information did not influence drinking intentions for the subsequent week.

**Conclusions:**

Alcohol consumers allocate minimal attention to warning labels on alcohol packaging and even if their attention is directed to these warning labels, this has no impact on their drinking intentions. The lack of attention to warning labels, even among people who actively want to cut down, suggests that there is room for improvement in the content of health warnings on alcohol packaging.

**Electronic supplementary material:**

The online version of this article (doi:10.1186/s12889-017-4055-8) contains supplementary material, which is available to authorized users.

## Background

In March 2011, alcohol beverage companies in the UK pledged to put warning labels on 80% of alcoholic drink containers as part of the public health responsibility deal [1]. These labels contain 1) the alcohol content (UK units), 2) the daily guidelines for maximum alcohol consumption, 3) a pregnancy warning, 4) a link to drinkaware.co.uk, the website of an industry sponsored charity (optional), and 5) a responsibility statement (optional; [2]).^1^ Warning labels have a limited effect on drinking behaviour. Narrative reviews of the evidence on alcohol health warnings demonstrated that public awareness of the warning label typically increases after implementation, but this does not translate to increased alcohol-related risk perceptions or reduced alcohol consumption [3–5]. Similarly, a systematic review showed that information-based policies (such as warning labelling) are generally ineffective [6], and researchers have argued that the pledges included in the responsibility deal are therefore unlikely to affect behaviour [7].

It is possible that warning labels have a limited effect on drinking behaviour because people pay little attention to them. Indeed, participants spent on average 7% of total viewing time looking at warning messages in alcohol advertisements [8]. However, there are likely to be individual differences in the amount of attention paid to health warning information, which may be important. Tobacco and food literature shows that consumption habits [9, 10] and goals [11] affect attention towards warning labels. In turn, attention to warning labels might also influence behaviour. For example, bar visitors drank less alcohol if their attention had been drawn to warning labels [12]. Similarly, nutrition labels had a stronger influence on product choice when they were attended to longer [13]. This raises the possibility that if warning labels on alcohol packaging are sufficiently ‘attention grabbing’, their impact on alcohol consumption at the population level could be substantial.

Unfortunately, nothing is known about the extent to which alcohol consumers attend to warning labels, how much their attention is related to individual differences in drinking behaviour and motivation to change it, and whether beneficial behaviour change is a likely consequence of increasing attention to warning labels on packaging. The purpose of the studies reported here was to investigate how much attention is paid to warning labels and branding on alcohol beverage containers, and how individual differences in this are associated with individual differences in drinking behaviour and motivation to change it. In both studies, we measured participants’ eye movements towards brand information and warning labels whilst they viewed alcohol beverage containers. Study 1 was an exploratory study that gathered descriptive information about how much attention alcohol consumers pay to health information and investigated correlations between attention and drinking habits. We hypothesized that heightened motivation to reduce drinking would be associated with increased attention to health warnings. In study 2, we experimentally manipulated motivation to reduce drinking and attention to health warnings in order to investigate the causal relationships between them.

## Study 1

### Method

#### Participants

Sixty participants (63% female) were recruited via online advertisements circulated among students and staff of the University of Liverpool. The sample size was based on previous research on attention to warning labels in alcohol print advertisements [8]. Participants were eligible to take part if they were aged over 18 and did not wear glasses. The majority were alcohol consumers (*n* = 58). Their mean age was 21.27 (SD = 3.61). They had a mean Alcohol Use Disorders Identification Test (AUDIT) score of 10.67 (SD = 6.54) and drank on average 32.12 (SD = 29.15) UK units in the 14 days prior to the experiment (1 UK unit = 8 g of alcohol). The study received ethical approval from the University of Liverpool Research Ethics Committee.

#### Materials

##### Stimuli

We photographed 50 beverage containers (bottles or cans) of various brands and types of alcoholic and non-alcoholic beverages that included health/warning labels (i.e., UK warning label on alcohol containers, nutrition information on soda containers). We photographed 25 alcohol containers (11 bottles/cans of beer, 6 cans of pre-mixed cocktails, 3 bottles/cans of cider, 3 bottles of alcopops and 2 bottles of wine) and 25 soda containers (23 bottles/cans of carbonated soft drinks and 2 bottles of fruit juice). We took four photographs of each container, two of the front and two of the back. One front and one back picture depicted the whole bottle or can, whereas a different picture depicted a close-up of the front label and the back label. The location of the alcohol warning labels varied between the containers. All aspects of the warning labels were visible and readable in the close-up during the viewing task. Most warning labels were in compliance with the guidelines specified in the responsibility deal and included the alcohol content, the daily guidelines for alcohol consumption, a pregnancy warning, an optional link to drinkaware.co.uk, and an optional responsibility statement. Two labels also included nutrition information. Three labels did not meet the minimum requirements: they did not include the daily recommended guidelines, and two of these also did not include a pregnancy warning. Nevertheless, we included these labels in our analyses as research has shown that 22.4% of alcohol warning labels did not comply with the responsibility deal guidelines [14]. Therefore, our stimuli were representative of the warning labels used in the UK.

##### Eye-tracker task

Participants were asked to view images of beverage containers (viewing phase) before their memory for the containers was tested (recognition phase; the latter was included to encourage participants to pay close attention during the viewing phase). In the viewing phase participants viewed 40 containers from the stimulus set (20 alcohol, 20 soda). They were instructed to use the arrow keys to manipulate the display of the containers. The left and right arrow keys were used to alternate between front and back. The up arrow was used to zoom in on the label and the down arrow was used to zoom out. Each container was presented for 15 s and participants were free to manipulate the presentation of the container in any way they liked. Whether the ‘zoomed out’ front or back of the container was presented first was randomized on a trial-by-trial basis. To ensure that all participants had the same starting position at image onset, participants were instructed to look at a fixation cross that was presented for 1 s before the trial started. Participants’ eye movements were measured using an ASL Eye-Trac D6 (Applied Science Laboratories, Bedford, MA) at a sampling rate of 120 Hz.

In the recognition phase, participants were shown a second set of 20 images (10 new and 10 of the 40 that had been presented during the viewing phase) and were asked to indicate whether or not each image had been present in the previous set by pressing a “yes” or “no” button. Recognition accuracy was defined as the percentage of correct trials. Participants correctly answered *M* = 95.83% (SD = 5.38) of the recognition trials.

#### Questionnaires

##### Alcohol use disorders identification test (AUDIT [15])

The AUDIT is a 10-item screening instrument assessing hazardous patterns of alcohol use and dependence symptoms. An example of an item is “How often do you have six or more drinks on one occasion?”. Each item is answered in a multiple choice format (e.g. “never”, “less than monthly”, “monthly”, “weekly” or “daily or almost daily”). Scores range between 0 and 40. AUDIT scores of 8 or higher are indicative of hazardous or harmful drinking patterns [15]. The AUDIT has good test-retest reliability, internal reliability and construct validity [16].

##### 14-day retrospective timeline follow-back diary (TLFB [17])

Participants were required to sum up for every day of the past two weeks, how many alcoholic drinks they had consumed in UK units. The TLFB has high test-retest reliability and good concurrent validity [17, 18].

##### Temptation Restraint Inventory – Restrain subscale (TRI [19])

The TRI restraint subscale is a 3-item scale answered on a 9-point Likert scale with anchors “never” and “always”. An example of an item is “How often do you attempt to cut down the amount you drink?”. Scores on the TRI restrain subscale range between 3 and 21. The TRI has adequate internal reliability and concurrent validity [19].

##### Readiness to Change Questionnaire (RTCQ [20])

The RTCQ is a questionnaire with three subscales (Precontemplation, Contemplation, and Action). The subscales are 4-item scales answered on a 5-point Likert scale with anchors “strongly disagree” and “strongly agree”. Examples of items are “I don’t think I drink too much” (precontemplation subscale), “I enjoy my drinking, but sometimes I drink too much” (contemplation subscale), and “I am trying to drink less than I used to” (action subscale). Scores on each RTCQ subscale range between−8 and 8. The RTCQ has good internal reliability and concurrent validity [20]*.*

##### Contemplation ladder [21]

The contemplation ladder is an 11-point scale on which participants are required to indicate their readiness to reduce their drinking (ranging from 0 “No thought of reducing how much I drink per occasion” to 10 “Taking action to reduce the number of drinks I have per occasion”). The contemplation ladder has good concurrent validity [21].

##### Dutch Eating Behaviour Questionnaire – Restraint subscale (DEBQ; [22])

Dietary restraint was measured with the DEBQ Restraint subscale. This is a 10-item scale answered in a multiple choice format (“not relevant”, “never”, “seldom”, “sometimes”, “often”, “very often”). An example of an item is “Do you watch exactly what you eat?”. Scores on the DEBQ Restraint subscale range between 10 and 50. The DEBQ Restraint subscale has high internal reliability and test-rest reliability [23] and good construct validity [24].

#### Procedure

After providing informed consent, participants completed the eye-tracker task. Then, they completed the questionnaire battery on a computer. A motivation to reduce drinking score was created by averaging the TRI restraint subscale, the RTCQ contemplation and action subscales and the contemplation ladder as these scales were strongly correlated (*r* = .53–.80, *p*s < .001). Finally, participants were thanked and debriefed. Participants received study credits or a £5 shopping voucher.

#### Data preparation and analysis

On each container, Areas of Interest (AOIs) were created by assigning the warning label and any calorie information to the category Health; any brand information, such as the logo and any brand messages to the category Brand; and everything else (e.g., barcode, recycling logo, blank packaging material) to the category Rest. The relative size of each AOI was calculated by dividing the number of pixels in the area by the total number of pixels of the container. The complexity of each AOI was calculated by dividing the compressed file size by the uncompressed file size [25]. Brightness and contrast values for each AOI were obtained using GNU Imagine Manipulation Program 2.

The different containers varied considerably in their visual characteristics (see Table 1), and conventional multivariate statistics are unable to control for this within-stimulus variability. Therefore, we used multilevel modelling to analyse eye movements. Data were organised in three levels, with AOIs (Brand, Health, Rest; level 1) nested in individual containers (40 containers; level 2) nested in data from each individual participant (level 3). To eliminate noise due to inaccurate eye-tracking, trials in which participant spent less than 50% of the viewing time looking at the product (Health, Brand and Rest combined – the only stimuli on the screen) were excluded from the analyses (12%). This percentage is similar to previous research on visual attention to tobacco warning labels, in which 8% [9] to 14% [26] of participants were excluded from analyses due to inaccurate tracking.

**Table 1:** Studies 1 and 2. Stimulus characteristics

	Alcohol (*n* = 20)			Soda (*n* = 20)		
	Brand	Health	Rest	Brand	Health	Rest
Variable	*M (SD*)	*M* (*SD*)	*M* (*SD*)	*M* (*SD*)	*M* (*SD*)	*M* (*SD*)
Size (% of total container)	34.24 (12.56)^a^	4.25 (3.38)^b^	61.51 (15.04)^c^	25.10 (10.90)^d^	5.47 (2.88)^b^	69.42 (12.21)^c^
Complexity (compression ratio)	.22 (.04)^a^	.27 (.06)^b^	.10 (.02)^c^	.26 (.03)^d^	.22 (.07)^d^	.12 (.02)^e^
Brightness (average luminosity)	111.92 (44.07)^a^	105.78 (53.48)^a^	91.54 (37.22)^a^	128.21 (36.87)^a^	118.19 (48.83)^a^	107.45 (38.19)^a^
Contrast (luminosity variance)	59.07 (11.71)^a^	48.58 (15.46)^b^	54.22 (12.33)^a^	59.07 (11.71)^a^	46.79 (11.92)^b^	52.70 (13.05)^a^

Note: Comparisons are between means in the same row. Different superscripts indicate a significant difference between means (*p* < .05)

We created multilevel models to analyse the effect of stimulus characteristics and drinking habits on fixation time. AOI (brand, health, rest (reference category, dummy coded)), order of presentation, size, complexity, brightness, and contrast were level 1 predictors; picture type (alcohol, soda (reference category)) was a level 2 predictor; and motivation to reduce drinking, alcohol consumption, AUDIT scores and dietary restraint were level 3 predictors. The models included random intercepts for all three levels.

In Model 1, we included all level 1 and level 2 predictors and their first and second order interactions with AOI and picture type. Model 1 showed that stimulus characteristics significantly influenced attention to the different AOIs on alcohol and soda packaging. In Model 2, we included all level 3 predictors and their first and second order interactions with AOI and picture type. A chi-squared test showed that Model 2 was a significantly better fit than Model 1 (*χ* ^2^(24) = 1015.93, *p* < .001), indicating that both participant characteristics and stimulus characteristics predicted fixation time.

### Results

#### Attention to branding and health warnings (Fig. 1)

**Fig. 1:**
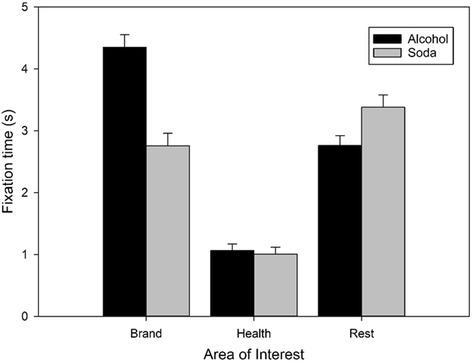
Visual attention to the different areas of interest (Brand, Health, Rest) on alcohol and soda packaging. Bars represent raw mean fixation time (s) averaged out across trials. Error bars indicate SEM

Over the 15 s viewing period, participants looked at alcohol warning labels for 1.03 s (*SD* = 0.89, 7%). A drink type (alcohol, soda) × AOI (brand, health, rest) repeated measures ANOVA revealed significant main effects of drink type (*F*(1, 56) = 63.97, *p* < .001, η^2^_p_ = .53) and AOI (*F*(2, 112) = 84.47, *p* < .001, η^2^_p_ = .60) that were qualified by a significant interaction (*F*(2, 112) = 71.09, *p* < .001, η^2^_p_ = .56). Post-hoc comparisons showed that participants spent less time viewing health information than brand information (alcohol t(58) = 14.36, *p* < .001, *d* = 1.87; soda t(56) = 7.17, *p* < .001, *d* = 0.95) and the rest of the packaging (alcohol t(58) = 10.62, *p* < .001, *d* = 1.38; soda t(56) = 12.95, *p* < .001, *d* = 1.71). Participants also looked longer at alcohol branding than the rest of the packaging, t(58) = 6.73, *p* < .001, *d* = 0.88, but less long at soda branding than the rest of the packaging, t(56) = 2.21, *p* = .03, *d* = 0.29. Participants attended more to alcohol than soda branding, t(56) = 11.78, *p* < .001, *d* = 1.56, and less to the rest of alcohol than soda packaging, t(56) = 4.44, *p* < .001, *d* = 0.59, but spent similar amounts of time viewing health warnings on alcohol and soda products, t(56) = .91, *p* = .37, *d* = 0.12.

#### Stimulus characteristics

The multilevel models revealed that the visual characteristics of branding and warning labels significantly affected attention. Alcohol warning labels were attended to longer when they were larger in size and less complex (see Additional file 1 for discussion).

#### Individual differences

Model 2 revealed a significant motivation to reduce drinking × AOI brand (vs health and rest) × picture type interaction (see Table 2). Motivation to reduce drinking was negatively associated with attention to branding on alcohol packaging. There was also a significant motivation to reduce drinking × AOI health (vs brand and rest) × picture type interaction: motivation to reduce drinking was negatively associated with attention to health warnings on alcohol packaging. Taken together, these results indicate that participants high in motivation to reduce drinking paid less attention to alcohol branding and health warnings and more attention to the rest of the packaging. Recent alcohol consumption and AUDIT scores were not significant predictors of attention. There was a significant association between dietary restraint and attention to branding, which is discussed in Additional file 2.

**Table 2:** Study 1. Multilevel regression model including stimulus-level and participant-level predictors. Area of Interest (AOI; Brand, health, rest, dummy coded with rest as reference category), brightness, contrast, complexity, and size were level 1 (AOI-level) predictors. Picture type (Alcohol, soda, dummy coded with soda as reference category) and presentation order were level 2 (Picture level) predictors. AUDIT scores, recent alcohol consumption, motivation to reduce drinking and dietary restraint were level 3 (Participant level) predictors. All predictors were included as individual main effects and in all possible two-way and three-way interactions with picture type and AOI

Variable	Model 2 (stimulus-level and participant-level predictors)					
		Two-way interactions			Three-way interactions.	
	Main effect	× Picture type	× AOI brand	× AOI health	× AOI brand × Picture type	× AOI health × Picture type
	*b* (*SE*)	*b* (*SE*)	*b* (*SE*)	*b* (*SE*)	*b* (*SE*)	*b* (*SE*)
Intercept	3.22 (0.84)	-	-	-	-	-
AOI brand	−3.73 (1.20)**	0.88 (1.72)	-	-	-	-
AOI health	−2.95 (0.97)**	0.18 (1.29)	-	-	-	-
Picture type	−0.61 (1.09)	-	0.88 (1.72)	0.18 (1.29)	-	-
Order	0.01 (0.01)	−0.01 (0.01)*	0.00 (0.01)	−0.003 (0.01)	0.01 (0.01)	0.01 (0.01)
Brightness	−0.001 (0.002)	−0.001 (0.003)	0.002 (0.003)	0.001 (0.003)	0.01 (0.01)	0.002 (0.004)
Contrast	−0.01 (0.01)*	0.01 (0.01)	0.01 (0.01)	0.01 (0.01)	−0.01 (0.01)	0.01 (0.01)
Complexity	11.12 (4.18)**	−1.90 (5.98)	−8.624 (4.83)^+^	−10.46 (4.27)*	2.41 (6.62)	−0.76 (6.13)
Size	−0.01 (0.01)	0.01 (0.01)	0.02 (0.01)*	0.08 (0.03)*	0.02 (0.04)	−0.02 (0.01)^+^
AUDIT	−0.004 (0.02)	0.01 (0.02)	0.03 (0.02)	0.004 (0.02)	−0.04 (0.03)	−0.004 (0.03)
Alcohol consumption (last 14 days)	0.002 (0.01)	−0.004 (0.01)	−0.002 (0.01)	−0.01 (0.01)	0.003 (0.01)	0.01 (0.01)
Motivation to reduce drinking	−0.04 (0.03)^+^	0.08 (0.04)**	0.01 (0.03)	0.05 (0.03)^+^	−0.08 (0.04)**	−0.09 (0.04)*
Dietary restraint	−0.02 (0.01)*	−0.02 (0.01)	0.02 (0.01)*	0.01 (0.01)	0.02 (0.01)	0.02 (0.01)
Random effects	Residual variance		Proportion residual variance explained			
Level 3	0.14 (0.03)		1.46%			
Level 2	3.18 (0.06)		17.15%			
Level 1	0 (0)		-			
*χ* ^2^(24)	894.27***					

Note: ^+^ *p* < .10, * *p* < .05, ** *p* < .01, *** *p* < .001

## Study 2

In study 2, we investigated the causal relationship between motivation to reduce drinking and attention allocation to branding/health warnings. First, to manipulate motivation to reduce drinking participants received a brief intervention regarding their drinking, or a control intervention. As the brief intervention predominantly targets people who drink in excess of the UK drinking guidelines, we recruited heavy drinkers. After the intervention, we measured attention to alcohol packaging. We hypothesized that participants would pay more attention to warning labels (and less to branding) after the alcohol intervention than the control intervention. Second, we manipulated attention to alcohol packaging so that participants either had to attend to warning labels or brand information. We used drinking intentions as the outcome measure, because they predict consumption [27] and are affected by changes in motivation to reduce drinking [28]. We hypothesized that participants who attended to health warnings would intend to drink less in the subsequent week than those who attended to branding.

### Method

#### Participants

120 participants (65% female) were recruited via online advertisements circulated among students and staff at the University of Liverpool (see Table 3). They were eligible for participation if they were aged over 18, did not wear glasses and consumed more alcohol than the recommended UK guidelines (14 units/week for females, 21 units/week for males).^2^ There was no formal screening in place to check whether participants fulfilled these criteria prior to taking part, but the eligibility criteria were emphasized at multiple times prior to the start of the lab session. The study received ethical approval from the University of Liverpool Research Ethics Committee.

**Table 3:** Study 2. Participant characteristics for each advice condition (alcohol, control) and exposure condition (brand, health)

	Alcohol advice (*n* = 60)		Control advice (*n* = 60)	
	Brand exposure (*n* = 30)	Health exposure (*n* = 30)	Brand exposure (*n* = 30)	Health exposure (*n* = 30)
Variable	*M* (*SD*)	*M* (*SD*)	*M* (*SD*)	*M* (*SD*)
Gender (% female)	66.7%	60%	66.7%	66.7%
Age	24.27 (10.26)	22.27 (4.58)	25.33 (8.02)	25.10 (11.05)
AUDIT (α = .66)	13.37 (5.37)	13.33 (5.42)	13.10 (4.25)	13.07 (4.63)
Alcohol consumption (last 14 days)	48.13 (26.48)	51.23 (23.54)	47.00 (17.37)	48.27 (23.05)
Baseline motivation to reduce drinking (α = .81)	1.61 (3.64)^a^	2.90 (3.92)^a^	4.51 (2.95)^b^	2.64 (3.55)^a^
DEBQ Restraint (α = .92)	34.73 (8.08)	35.30 (10.48)	34.47 (8.93)	34.53 (10.92)

#### Materials

##### Stimuli

We used the same stimuli and questionnaires as in study 1. Because the contemplation ladder was administered after the manipulation, baseline motivation to reduce drinking was defined as the average of the TRI restraint scale and RTCQ contemplation and action subscales, which were strongly correlated (*r* = .52–.68, *p*s < .001).

##### Drinking intentions

To measure drinking intentions, participants were asked how many pints of cider/beer, large glasses of wine, and shots of hard liquor they intended to drink in the next week [29]. Their responses were combined into a single measure of intended consumption in UK units. Binge drinking intentions were measured with three 9-point Likert scales (e.g., “*Do you plan to binge*-*drink in the next week*?” [30]). The scores were averaged into a single binge drinking measure (α = .97).

##### Viewing task

The eye-tracker task was the same as in study 1, with the exception that participants only viewed 30 containers during the viewing phase (15 alcohol, 15 soda) and 12 containers in the recognition phase.

##### Screening and intervention programme for sensible drinking (SIPS) brief advice tool [31] and control

Participants were informed about their AUDIT scores and alcohol consumption, and the associated health risks, before receiving advice about population norms and the benefits of cutting down, followed by individualised tips to reduce their drinking. For the control condition, participants received brief advice on study habits. The advice closely followed the SIPS procedure, providing participants with information about different ways to study and their associated benefits and tailored tips to improve their own study habits (see Additional file 3).

##### Manipulation of attention task

Participants were informed that important information for the subsequent memory test would be highlighted. They viewed the back and front labels of 15 alcoholic drinks containers with a bright yellow border around either the warning label or the brand information. To manipulate attention, in the health exposure condition, the majority of the labels had a border around the warning label (13 labels, 86%), whereas in the brand exposure condition, the border was around the brand information.

#### Procedure

After giving consent, participants filled out the alcohol diary, AUDIT, TRI, and RTCQ. Then, half of the participants received brief advice on sensible alcohol consumption (alcohol advice condition), whilst the other half received brief advice about study habits (control condition). Then, participants did the viewing task. They were asked to indicate their motivation to reduce drinking on the contemplation ladder before and after the task. After this, participants received the manipulation of attention task. Half of the participants in the alcohol advice and control condition were allocated to the brand exposure condition and the other half were allocated to the health exposure condition. Allocation to the advice conditions and attention conditions was randomized. Then, participants completed the drinking intentions questionnaire and the DEBQ, followed by a bogus memory task to corroborate the stated aim of the manipulation of attention task. Finally, participants were thanked and debriefed. Participants received study credits or a £5 high street voucher.

#### Data preparation and analyses

We employed the same data preparation and analysis strategy for the viewing task as in study 1. Trials in which participants spent less than 50% of the viewing time looking at the stimuli were removed due to inaccurate tracking (9%). A model with the level 1 and level 2 predictors (Model 1) was compared with Model 2, which also included condition (alcohol advice vs control), and baseline motivation to reduce drinking as participant-level predictors (level 3). A chi-squared test showed that Model 2 was a significantly better fit than Model 1 (*χ* ^2^(12) = 31.72, *p* < .001), which indicates that the level 3 variables predicted fixation time above and beyond stimulus characteristics.

### Results

#### Baseline differences

A 2 (advice condition: alcohol, control) × 2 (exposure condition: brand, health) MANOVA with age, recent alcohol consumption, AUDIT scores and the baseline measure of motivation to reduce drinking as dependent variables revealed significant baseline differences between conditions. There were significant group differences in motivation to reduce drinking for the advice conditions (*F*(1, 116) = 4.20, *p* = .04, η^2^_p_ = .04), which were qualified by a significant advice × exposure condition interaction on motivation to reduce drinking (*F*(1, 116) = 5.99, *p* = .02, η^2^_p_ = .05). Post-hoc t-tests showed that participants in the control condition had a stronger baseline motivation to reduce drinking than participants in the alcohol advice condition, *t*(118) = 2.02, *p* = .046, *d* = .37. This difference between advice conditions was only significant among participants in the brand exposure condition, *t*(58) = 3.39, *p* = .001, *d* = .88, and not among those in the health exposure condition, *t*(58) = .27, *p* = .79, *d* = .07, see Table 3. There were no significant baseline differences in age, recent alcohol consumption, and AUDIT scores (all *p*s > .23).

#### Free viewing

##### Manipulation check

An independent samples *t*-test revealed no significant difference in contemplation ladder scores between the alcohol advice condition (*M* = 4.72, *SD* = 2.82) and the control condition (*M* = 3.95, *SD* = 2.94), *t*(118) = 1.46, *p* = .15, *d* = .27. Therefore, the SIPS manipulation did not increase motivation to reduce drinking.

##### Effects of advice condition on free viewing (Fig. 2)

**Fig. 2:**
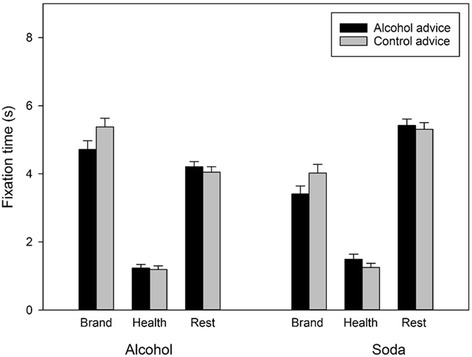
Effect of condition (alcohol advice, control) on visual attention to AOIs (brand, health, rest) on alcohol and soda packaging. Alcohol advice reduced attention to branding on alcohol and soda packaging. Bars represent raw mean fixation time (s) averaged out across trials. Error bars indicate SEM

Over a 15 s viewing period, participants looked at alcohol warning labels for 1.20s (*SD* = 0.81, 8%). There was a significant AOI brand × condition interaction, indicating that participants who received alcohol advice spent less time viewing brand information than those in the control condition (see Table 4). The non-significant AOI brand × picture type × condition interaction showed that the relation between condition and attention to branding did not depend on picture type (alcohol vs. soda). The AOI health × condition and the AOI health × picture type × condition interactions were non-significant. This indicates that participants who received alcohol advice did not compensate their reduced attention to branding by increasing attention to health warnings on alcohol or soda containers, but instead increased their attention to the rest of the packaging.

**Table 4:** Study 2. Multilevel regression model including stimulus-level and participant-level predictors. Area of Interest (AOI; Brand, health, rest, dummy coded with rest as reference category), brightness, contrast, complexity, and size were level 1 (AOI-level) predictors. Picture type (Alcohol, soda, dummy coded with soda as reference category) and presentation order were level 2 (Picture level) predictors. Advice condition (Alcohol, control, dummy coded with control as reference category) and baseline motivation to reduce drinking were level 3 (Participant level) predictors. All predictors were included as individual main effects and in all possible two-way and three-way interactions with picture type and AOI

Variable	Model 2 (stimulus-level and participant-level predictors)					
		Two-way interactions			Three-way interactions.	
	Main effect	x picture type	x AOI brand	x AOI health	x AOI brand x picture type	x AOI health x picture type
	*b* (*SE*)	*b* (*SE*)	*b* (*SE*)	*b* (*SE*)	*b* (*SE*)	*b* (*SE*)
Intercept	1.78 (1.45)	-	-	-	-	-
AOI brand	−0.26 (2.14)	−22.46 (3.04)***	-	-	-	-
AOI health	−0.54 (1.72)	−8.46 (2.23)***	-	-	-	-
Picture type	8.46 (1.93)***	-	22.46 (3.04)***	−8.46 (2.23)***	-	-
Order	−0.01 (0.01)	0.02 (0.02)	−0.02 (0.02)	0.01 (0.02)	−0.002 (0.02)	−0.01 (0.02)
Brightness	0.005 (0.003)+	−0.03 (0.01)***	−0.01 (0.01)	−0.01 (0.01)	0.10 (0.01)***	0.04 (0.01)***
Contrast	−0.01 (0.01)	−0.15 (0.02)***	0.003 (0.01)	0.01 (0.01)	0.16 (0.02)***	0.15 (0.02)***
Complexity	10.98 (7.94)	23.85 (11.08)*	−4.14 (9.06)	−10.41 (8.20)	−18.89 (12.32)	−30.03 (11.36)**
Size	0.03 (0.01)**	0.01 (0.01)	0.04 (0.02)*	−0.02 (0.06)	0.14 (0.03)***	0.20 (0.07)**
Advice condition	0.03 (0.27)	0.37 (0.32)	−0.69 (0.29)*	0.11 (0.29)	−0.45 (0.40)	−0.51 (0.40)
Baseline motivation to reduce drinking	−0.01 (0.04)	0.04 (0.04)	−0.02 (0.04)	0.03 (0.04)	−0.07 (0.06)	−0.06 (0.06)
Random effects	Residual variance		Proportion residual variance explained			
Level 3	0.55 (0.12)		−29.67%			
Level 2	4.29 (0.26)		12.76%			
Level 1	16.97 (0.29)		35.1%			
*χ* ^2^(47)	31.72**					

#### Exposure task

##### Manipulation check

Participants in the brand attention condition fixated longer on brand (*M* = 2.41, *SD* = 1.21) than health information (*M* = 1.05, *SD* = 0.42) and participants in the health attention condition fixated longer on health (M = 2.13, *SD* = 1.05) than brand information (M = .86, *SD* = .53; *F*(1,116) = 133.24, *p* < .001, η^2^_p_ = .58). Therefore, the manipulation of attention was successful.

##### Effect of attention to brand and health information on drinking intentions

A 2 (exposure; brand, health) by 2 (condition; alcohol advice, control) MANOVA with binge drinking intentions and intended consumption as the DVs showed that exposure did not significantly affect drinking intentions (Multivariate *F*(2, 115) = .47, *p* = .62, η^2^_p_ = .01). Neither did condition (Multivariate *F*(2, 115) = 1.94, *p* = .15, η^2^_p_ = .03), or the interaction between exposure and condition (Multivariate *F*(2,115) = .64, *p* = .53, η^2^_p_ = .01).

#### General discussion

In two studies, we investigated alcohol consumers’ attention to warning labels on alcohol packaging, and how this is associated with individual differences in motivation to reduce drinking. The results showed that people paid minimal attention to warning labels on alcohol packaging (7–8% of total viewing time). In study 1, we demonstrated that self-reported motivation to reduce drinking reduced attention to both branding and warning labels on alcohol packaging. Although we did not replicate this association in study 2, we did demonstrate that a brief alcohol intervention reduced attention to branding, although this effect was not specific for alcohol packaging and the brief alcohol intervention did not influence participants’ motivation to reduce drinking. Contrary to hypotheses, our experimental manipulation that encouraged participants to focus their attention on warning labels did not affect their drinking intentions for the subsequent week.

A possible explanation is that participants do not particularly notice warning labels, due to their current design [14, 32]. Our results show that alcohol warning labels on average take up less than 5% of the packaging and that attention to warning labels is roughly proportional to their size. Additionally, our results suggest that large alcohol warning labels attracted more attention, but we did not experimentally test this. Research regarding tobacco labels supports this: larger labels increased message recall compared to smaller labels [33]. Another explanation is that participants do not see the current warning label as goal-relevant. This might be because it does not show the consequences of exceeding the recommended guidelines. Additionally, research suggests that “drink responsibly” messages (as included in the UK warning labels) are primarily used as a means to promote drinking [34–36] rather than raise awareness of the harmful consequences of alcohol consumption. Therefore, participants who are motivated to reduce drinking might view them as another part of the product branding, and subsequently avoid them. Indeed, some researchers argue that alcohol warning labels should be more like tobacco warnings and nutrition labels and provide clear information about alcohol-related risks and unambiguous behavioural recommendations in order to increase their effectiveness [37, 38].

Indeed, Al-hamdani and Smith [39] demonstrated that warning labels that provided unambiguous information about the effect of alcohol consumption on liver cancer made people perceive the product more negatively compared to non-labelled products. Similarly, warning labels about cancer also increased participants’ intentions to reduce drinking [40, 41] and reduced participants’ drinking speed [42], regardless of whether the warning label was text-only or included a picture of liver cancer. Another recent study showed that the inclusion of a self-affirming implementation intention in addition to the standard UK warning label reduced alcohol consumption at one month follow-up [43]. Research on the effect of alcohol warning advertisements demonstrated that exposure to warnings affected urge to drink via increased negative affect in response to the warnings [44]. This suggests that alcohol warning labels might need to elicit negative emotions in order to reduce consumption. Future research should explore the effect of label design and content on attention. Increasing the visual salience of warning labels by using plain packaging [9, 10], graphic warnings [26] and front-of-pack labelling [45] might be more effective in attracting and maintaining attention, as shown in tobacco and food research.

These studies have some limitations. The viewing task in both studies comprised a 15 s viewing period for each beverage container and it was framed as a memory task to ensure that participants would attend to the packaging. The length of exposure and the instructions might have increased attention to areas that participants would normally ignore. Additionally, the alcohol advice manipulation did not increase motivation to reduce drinking, which means that the significant effect of advice condition cannot be interpreted as an effect of motivation to reduce drinking. Finally, when viewing multiple products at the same time, people pay more attention to the product they prefer [46]. We did not measure brand preferences in these studies, so it is possible that individual differences in brand preferences affected attention allocation to the branding/health warnings. However, we showed each product by itself, so there was no competition for attention between brands. Additionally, everyone had to view each product for exactly 15 s, so participants could not decide to view the product for a shorter amount of time if they did not prefer the brand. Therefore, it is unlikely that individual differences in brand preferences had a substantial effect on our results. Our study also had strengths. We used existing alcohol containers with current UK health warnings and used multilevel modelling to control for differences in packaging design. We also used a combination of correlational and experimental designs to investigate the relation between motivation to reduce drinking and attention. Additionally, we allowed participants to manipulate their view of the beverage containers (front/back, zoomed in/out) in any way they liked, which is more similar to real life viewing conditions. However, it should be noted that the manipulation of the containers was not the same as participants handling the container, which would have allowed them to tilt the container in order to better view vertical labels, for example.

### Conclusions

To conclude, our studies show that people pay minimal attention to current UK warning labels on alcohol packaging. Motivation to reduce drinking decreases attention to branding, but does not increase attention to warning labels. Drinking intentions were not affected by attention to warning labels, even when participants had to attend to them. Changes in warning label design that make the label more visually salient and content are advised.

## Additional files

Additional file 1: Study 1 – visual characteristics. A discussion of the association between stimulus size and complexity and visual attention to warning labels. (DOCX 12 kb)
Additional file 2: Study 1 – dietary restraint. A discussion of the association between dietary restraint and visual attention to branding. (DOCX 18 kb)
Additional file 3: Control intervention. Stimulus material used as part of the control invention regarding study habits. (PDF 410 kb)

## Abbreviations

AOI: Area of Interest
AUDIT: Alcohol Use Disorders Identification Test
DEBQ: Dutch Eating Behaviour Questionnaire
RTCQ: Readiness to Change Questionnaire
SIPS: Screening and intervention program for sensible drinking
TRI: Temptation Restraint Inventory

## Footnotes

1 One might argue that these labels do not warn against any adverse outcomes and should therefore not be called warning labels. In previous literature, these types of “drink responsibly” labels have been referred to as “warning labels” or “health warnings” [47]. For the sake of consistency, we will refer to the drinkaware labels as health warning labels in this manuscript.

2 Recruitment for this study took place from May 2014 – September 2015, before the UK guidelines were revised (January 2016).
