# Supplementary material for: Alcohol consumers’ attention to warning labels and brand information on alcohol packaging: Findings from cross-sectional and experimental studies
Source: BMC Public Health. 2017 Jan 26;17:123. doi: 10.1186/s12889-017-4055-8 (PMC5267428; doi:10.1186/s12889-017-4055-8)
Supplement: Additional file 1: — Study 1 – visual characteristics. A discussion of the association between stimulus size and complexity and visual attention to warning labels. (DOCX 12 kb) [file 12889_2017_4055_MOESM1_ESM.docx]

**Study 1 – visual characteristics**

*Stimulus size*

The multilevel model revealed a significant size × AOI health (vs brand and rest) interaction (b = 0.08, SE = 0.03, p = .01) and a nonsignificant size × AOI health × picture type interaction (b = -0.02, SE = 0.04, p = .10), indicating that larger health warnings were attended to longer, regardless of picture type. The size × AOI brand (vs health and rest) interaction (b = 0.02, SE = 0.01, p = .05) was significant, and the size × AOI brand × picture type interaction was not significant (b = 0.02, SE = 0.01, p = .60), indicating that larger branding attracted more attention, regardless of picture type.

*Stimulus complexity*

The multilevel model revealed a significant complexity × AOI health interaction

(*b* = -10.46, SE = 4.23, *p* = .01) and a non-significant complexity × AOI health × picture type interaction (*b* = -0.8, SE = 6.13, *p* = .90), indicating that more visually complex health warnings were attended to less, regardless of picture type. The complexity × AOI brand interaction (*b* = -8.62, SE = 4.83, *p* = .07) was marginally significant, and the complexity × AOI brand × picture type interaction was non-significant (*b* = 2.41, SE = 6.62, *p* = .72), indicating that more complex branding attracted less attention, regardless of picture type.
