# Supplementary material for: Alcohol consumers’ attention to warning labels and brand information on alcohol packaging: Findings from cross-sectional and experimental studies
Source: BMC Public Health. 2017 Jan 26;17:123. doi: 10.1186/s12889-017-4055-8 (PMC5267428; doi:10.1186/s12889-017-4055-8)
Supplement: Additional file 2: — Study 1 – dietary restraint. A discussion of the association between dietary restraint and visual attention to branding. (DOCX 18 kb) [file 12889_2017_4055_MOESM2_ESM.docx]

**Study 1**

*Dietary restraint*

The multilevel model revealed a significant dietary restraint × AOI brand interaction (*b* = 0.02, SE = 0.01, *p* = .03). Participants with higher dietary restraint fixated longer on branding. A non-significant dietary restraint × AOI brand × picture type interaction (*b* = 0.02, SE = 0.01, *p* = .11) showed that this relationship was regardless of picture type. The dietary restraint × AOI health interaction (*b* = 0.01, SE = 0.01, *p* = .16) and the dietary restraint × AOI health × picture type interaction (*b* = 0.02, SE = 0.01, *p* = .28) were non-significant. This indicates that participants high in dietary restraint did not compensate their decreased attention to branding by increasing attention to health warnings, but instead by increasing attention to the rest of the packaging, both for alcohol and soda containers.
