# Supplementary material for: Alcohol consumers’ attention to warning labels and brand information on alcohol packaging: Findings from cross-sectional and experimental studies
Source: BMC Public Health. 2017 Jan 26;17:123. doi: 10.1186/s12889-017-4055-8 (PMC5267428; doi:10.1186/s12889-017-4055-8)
Supplement: Additional file 3: — Control intervention. Stimulus material used as part of the control invention regarding study habits. (PDF 410 kb) [file 12889_2017_4055_MOESM3_ESM.pdf]

These are general study tips...

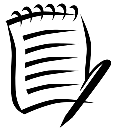
Take notes

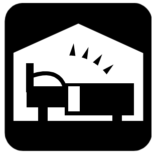
Sleep

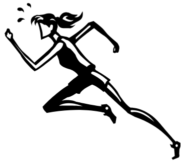
Exercise

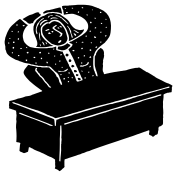
Take breaks

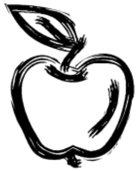
Eat healthy food

How many of these do you do?

...and each of these is a more specific adaptation.

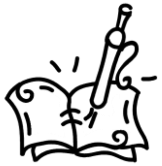
Pretend to take notes for someone else

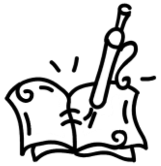
Test yourself

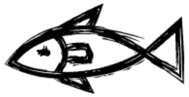
Eat oily fish

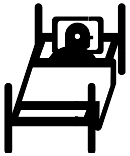
Sleep within 12 hours of studying

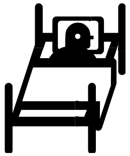
Study just before going to bed

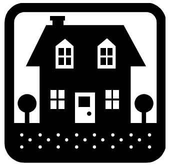
Study in different locations

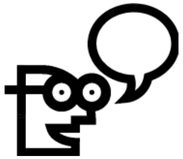
Read the information out loud

This table tells you what type of student you are.

Learning style	Characteristics		Specific tips
TACTILE LEARNER	Learns by touching and doing. Often speaks with their hands and may have difficulty sitting still.	Can easily remember things that were done, but may have difficulty remembering things they heard or saw.	• Chew gum<br>• Do hands-on activities whilst studying<br>• Draw
AUDITORY LEARNER	Stores information by the way it sounds. Often hums or talks to themselves when bored.	Understands spoken instructions better than written. Often learns by reading the material out loud.	• Record yourself<br>• Songs/rhymes<br>• Repeat<br>• Acronyms<br>• Read out loud<br>• Study with others
VISUAL LEARNER	Learns by reading or seeing pictures	Often closes eyes to remember things.	• Write down key words/ideas<br>• Draw pictures<br>• Avoid distractions while studying<br>• Colour code things<br>• Visualize things you see or hear

A **mnemonic** is a learning technique that translates information into a form that the brain can remember better than the original form.

You can make a mnemonic in **many different ways.** For example, creating a sentence with the same starting letters as the thing you want to remember (Please Excuse My Dear Aunt Sally).

It is recommended you get around 8 hours of **sleep** each night.

On top of that, losing **one night's sleep** can impair your reasoning and brain functioning for up to four days.

How do you feel?

You saw what type of learner you might be and received specific tips you can use to enhance your memory. **What do you think?**

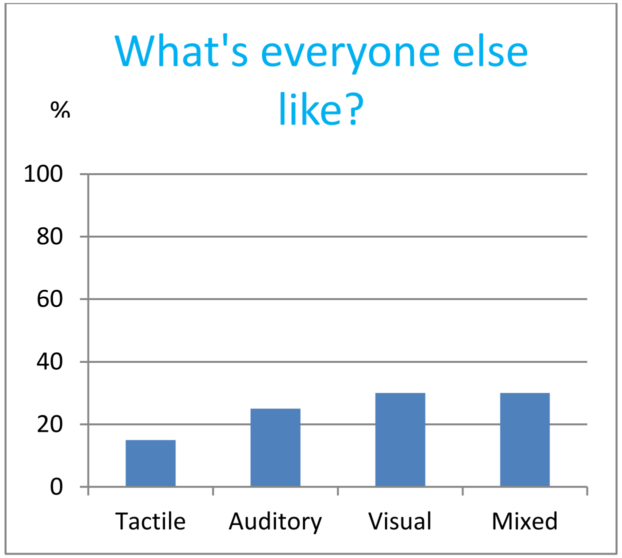

Making your plan

- Plan study sessions at those times you’re usually most active
- When bored, take a quick break or study a different subject
- Study in different locations
- Avoid studying when you’re tired
- Set realistic goals
- Limit your caffeine intake
- Eat plenty of fruit and vegetables

The benefits of starting now

- Takes time to change habits
- Applicable to many aspects of life, not just university
- Trains your brain
- Learn new skills more easily

What targets should you aim for?

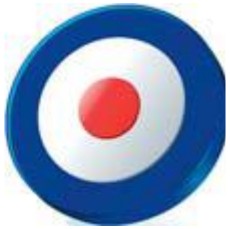

Study for 30 minutes every day

Sleep well

Find an interesting subject to learn to keep your brain active

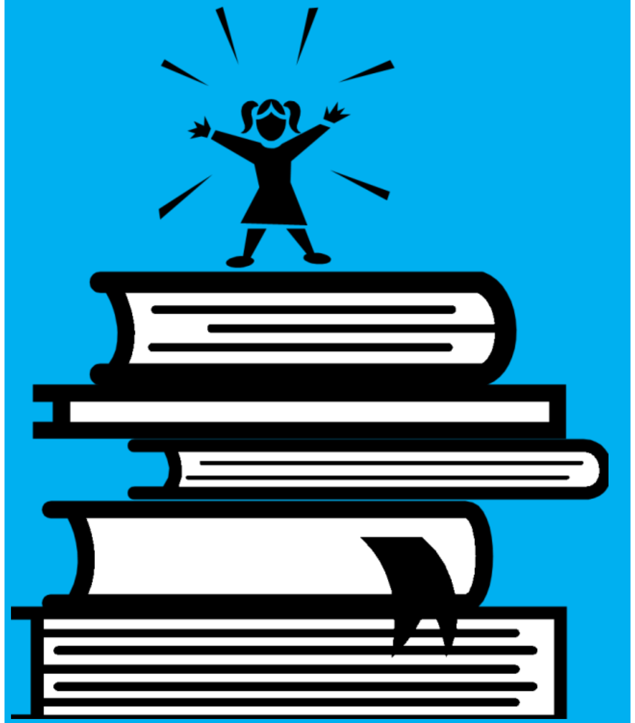

Brief advice about studying
